# A Liberal Compensation

**Published:** 1869-11

**Authors:** 


### A LIBERAL COMPENSATION.

We have had laid upon our table a door lock for dwellings, which promises to come into general favor.

This lock is burglar-proof, and cannot be opened without the key, hence, for sleeping rooms and such places as require security, this new invention is admirably adapted.

We have arranged with the inventor for such numbers as we may require, and propose to offer one lock to whoever shall send us a list of three subscribers to the Journal, with four dollars and a half.

As the best burglar-proof locks cost from three to ten dollars, and this is absolutely unpickable, and is easily adapted to any door, it is very valuable.

